# Sport and recreation for Indigenous peoples with neurodevelopmental disabilities in Canada: an empty scoping review and call to action

**DOI:** 10.3389/fresc.2026.1826523

**Published:** 2026-08-05

**Authors:** Grant Bruno, Stephanie Andreasen, Mariam Ahmad, Tierney Littlechild, Court Roy, Brianna Cuzzolino, Everett Sokol, Will Landon, Carly A. McMorris

**Affiliations:** 1 Samson Cree Nation (Treaty 6 Territory), Maskwacis, AB, Canada; 2 Department of Pediatrics, University of Alberta, Edmonton, AB, Canada; 3 School and Applied Child Psychology, Werklund School of Education, University of Calgary, Calgary, AB, Canada; 4 Owerko Centre, Alberta Children’s Hospital Research Institute, University of Calgary, Calgary, AB, Canada; 5 Faculty of Medicine and Dentistry, Women’s and Children’s Health Research Institute, University of Alberta, Edmonton, AB, Canada; 6 Ermineskin Cree Nation (Treaty 6 Territory), Maskwacis, AB, Canada; 7 Special Olympics Alberta, Edmonton, AB, Canada; 8 Special Olympics Canada, Toronto, ON, Canada; 9 Anishinaabe of Wauzhushk Onigum First Nation Wauzhushk Onigum First Nation, ON, Canada

**Keywords:** Canada, empty review, Indigenous, neurodevelopmental disabilities, recreation, scoping review, sport

## Abstract

Sport and recreation are central to physical health, social inclusion, and community well-being. Indigenous Peoples and individuals with neurodevelopmental disabilities (NDDs) both experience systemic barriers to participation; however, the intersection of these identities remains underexplored. This Indigenous-led scoping review examined the current state of published and grey literature on sport and recreation for Indigenous Peoples with NDDs in Canada and considered what research, policy, and practice gaps are evident in, or suggested by, this literature. Guided by PRISMA-ScR, comprehensive searches of academic and grey literature databases were conducted using terms related to Indigenous identity, neurodevelopmental disability, and sport or recreation. Using the PRISMA-ScR, two reviewers independently screened titles, abstracts, and full texts according to predefined inclusion criteria. Of 326 records identified, zero studies met eligibility criteria. The absence of eligible studies indicates a critical gap in research, policy, and programming. Indigenous Peoples with NDDs remain invisible within sport and recreation scholarship, underscoring the urgent need for Indigenous-led, culturally grounded, and inclusive research initiatives.

## Introduction

Sport and recreation play a critical role in promoting physical health, mental well-being, and social inclusion (1–3). However, Indigenous Peoples often encounter systemic barriers that limit their access to meaningful participation in these activities. Globally, Indigenous Peoples experience disproportionately high rates of disability due to the ongoing impacts of colonialism, historical trauma, and social determinants of health (4–6). Similarly, people with neurodevelopmental disabilities (NDDs) also face numerous obstacles to accessing sports and recreational activities, including personal barriers (e.g., lack of skill or coordination, preferences, fear of injury), social barriers (e.g., lack of friends, parental attitudes, negative attitudes of others), and environmental barriers (e.g., inadequate and inaccessible facilities, lack of transportation) (7). The intersection of NDDs with sport and recreation, particularly in Indigenous contexts, remains underexplored despite its connections with broader issues of marginalization, access to services, and the need for culturally responsive support systems.

Existing research on sports and recreation for individuals with disabilities has primarily focused on Western models of inclusion and adaptive programming, often neglecting the unique cultural, historical, and community-based perspectives of Indigenous Peoples (8). Similarly, research on Indigenous participation in sport has predominantly centred on elite competition, land-based practices, or youth engagement in Western sports, with minimal attention given to individuals with NDDs (9–11). Indigenous perspectives on disability are viewed through a holistic lens, emphasizing strengths, rather than solely focusing on deficits (12, 13). Understanding these cultural perspectives is crucial for developing sports programs that are inclusive and relevant to Indigenous Peoples with NDDs. Furthermore, this gap in the literature underscores the necessity for a comprehensive understanding of how Indigenous Peoples with NDDs experience and access physical activity through sports and recreation, as well as the cultural, structural, and systemic factors that influence participation. The ongoing effects of colonialism and historical trauma have resulted in compounded marginalization for Indigenous individuals with NDDs, creating significant barriers to accessing sports and recreation opportunities.

Terminology related to disability has evolved across various fields, including medicine, research, and education (14). It has increasingly engaged with marginalized populations to acknowledge the detrimental effects of specific language and to shift toward more inclusive and neuro-affirming terms (15). For the purposes of this article, we will use the term “Indigenous Peoples” to refer to individuals with an ancestral history of inhabiting a land from the earliest times or prior to the arrival of European colonists (16). Additionally, we will use the phrase “people (or individuals) with neurodevelopmental disabilities (NDDs)” to describe individuals who have conditions that arise during brain development and function, leading to challenges in learning, behaviour, or other developmental aspects that persist into adulthood (17).

Since time immemorial, Indigenous Peoples in Canada have participated in sports and recreational activities that are integral to their cultural identity, community, individual well-being, and resilience. Traditional activities such as lacrosse, canoeing, snowshoeing, and hunting-based competitions have historically served both practical and spiritual purposes, fostering social cohesion and facilitating the transfer of intergenerational knowledge (18). Despite facing systemic barriers, including colonial policies designed to suppress Indigenous physical and cultural practices, Indigenous athletes have consistently demonstrated remarkable resilience, excelling in both mainstream and Indigenous-led sporting environments (19).

Indigenous-led initiatives, such as the North American Indigenous Games (NAIG), have reclaimed sports as a vehicle for cultural revitalization, health promotion, and self-determination (11). In 2008, then Prime Minister Stephen Harper issued an apology for the harmful effects of the residential school system in Canada. Following this apology, the Truth and Reconciliation Commission (TRC) was established, emphasizing areas such as education, health, and justice as key aspects for improving health outcomes for Indigenous Peoples. Specifically, Calls to Action 87–91 outline the importance of inclusive, culturally responsive programming that supports the physical, mental, emotional, and spiritual well-being (20). However, despite these commitments, there remains a significant gap in addressing the needs of Indigenous individuals with NDDs whose inclusion in sport and recreation continues to be overlooked in research, policy, and practice.

Over the past several decades, the relationship between NDDs and sports and recreation has shifted from one of exclusion and segregation to an increase in inclusion and adapted opportunities (21). Historically, individuals with NDDs were often marginalized from organized sports due to societal misconceptions, a lack of accommodations, and medicalized perspectives that emphasized deficits rather than capabilities (22). Furthermore, early interventions primarily focused on educational physical activities, often conducted within institutional settings, rather than promoting participation in community settings or competitive sports (23, 24).The inaugural Special Olympics games in 1968 marked a pivotal moment, shifting perspectives to recognize the athletic potential of individuals with NDDs and advocating for their right to participate in sports and recreation (21). More recently, the neurodiversity movement, which emphasizes the inclusivity of all neurotypes, has influenced inclusive sports policies, emphasizing the need for strength-based approaches to self-determined participation (25).

Article 30 of the United Nations Convention on the Rights of Persons with Disabilities (UNCRPD) affirms the rights of all persons with disabilities to participate in recreation, leisure, and sport on an equitable basis with others (26). It calls upon nation-states and various levels of government to ensure that persons with disabilities have access to inclusive, adaptive, and accessible sporting activities at all levels. By centring the principles of dignity, non-discrimination, and full participation, the UNCRPD positions inclusive sport as a matter of equity and fundamental human rights. Applying these principles to Indigenous communities requires an understanding of cultural practices, traditional knowledge systems, and community-driven solutions. Inclusive sport and recreation programs for Indigenous Peoples with NDDs must reflect global human rights standards and Indigenous specific needs. Despite the progress made within the NDD space, barriers such as accessibility, stigma, and inequitable funding persist, necessitating continued efforts to ensure equitable approaches to sport and recreation for individuals with NDDs, particularly among Indigenous Peoples (27).

Despite related scholarship on Indigenous disability, Indigenous sport and recreation, and disability-inclusive sport, these bodies of literature have rarely been brought together to examine sport and recreation for Indigenous Peoples with NDDs in Canada (1, 5, 6, 8, 10–12, 21, 22, 27). This scoping review examined the current state of published and grey literature on sport and recreation for Indigenous Peoples with NDDs in Canada and considered what research, policy, and practice gaps are evident in, or suggested by, this literature. The guiding research question was: What is the current state of published and grey literature on sport and recreation for Indigenous Peoples with NDDs in Canada, and what research, policy, and practice gaps are evident in, or suggested by, this literature?

## Materials and methods

### Scoping review framework

Given the limited literature on the intersection of people with NDDs and Indigenous identity and their participation in sports and recreation, we conducted a scoping review to summarize the current literature. Our scoping review and data analysis were guided by the Preferred Reporting Items for Systematic Reviews and Meta-Analysis Extension for Scoping Reviews (PRISMA-ScR) framework (28). The search strategies were developed by the research team with guidance from a research librarian who specializes in health science research and conducting systematic and scoping reviews. Scoping reviews require two independent researchers (GB and SA) to review the articles separately. This review was guided by a working group of Indigenous (GB, TL, ES) and non-Indigenous (SA, CR, MA, BC, CM) members. A key feature of this review is that it was Indigenous-led, emphasizing the practice of relational accountability and collaboration. Relational accountability was upheld by ensuring ongoing dialogue and consensus-based decision-making among both Indigenous and non-Indigenous members of the research team throughout the review process.

### Positionality and Indigenous-led approach

Positionality is a critical component of ethical and reflexive research practices, particularly when engaging or working alongside Indigenous or marginalized communities (29). It recognizes that researchers are not objective or neutral actors; rather, they are influenced by their own identities, experiences, worldviews, and social locations (30). Including a positionality statement in a research article allows authors to situate themselves to the research context, participants, and knowledge systems involved. In the context of Indigenous-led research, positionality also serves as a commitment to relational accountability (31). By openly acknowledging their positionality, researchers contribute to more ethical, culturally responsive, and socially just research practices. As a First Nations scholar collaborating with both Indigenous and non-Indigenous colleagues, our positionalities shape how we engage with Indigenous communities, interpret traditional knowledge, and approach disability inclusion. We recognize the significance of reflecting on these identities to ensure respectful and accurate representation throughout the research process.

Dr. Grant Bruno is a nêhiyaw nâpêw (Plains Cree man) and a registered member of Nipishkopahk (Samson Cree Nation), an Assistant Professor in Pediatrics at the University of Alberta and is a parent of a child with an NDD. Stephanie Andreasen is a white woman and a graduate student at the University of Calgary, with over 15 years of experience supporting children and adults with NDDs and their families to foster inclusion within the community. Mariam Ahmad is a Pakistani settler, an Indigenous Health Research Coordinator at the Women's and Children's Health Research Institute at the University of Alberta, and the sibling of an adult with an NDD. Tierney Littlechild is a member of Samson Cree Nation and Community Research Coordinator for the Ispimihk Awâsisak (Sky Children) research program, which aims to support Indigenous child and youth wellbeing. Court Roy is a cisgender white woman and Manager, Partnership Programs at Special Olympics Alberta with eight years of experience supporting athletes with NDDs to access sport and recreation opportunities. Brianna Cuzzolino is a white queer woman and Manager, Programs at Special Olympics Canada with eight years' experience working in classrooms and sport and recreation programs for diverse youth and adults. Everett Sokol is a two-spirit member of the Métis Nation of Alberta and the Indigenous Engagement Coordinator with Special Olympics Alberta. Wabshkigaabo (Will Landon) is Anishinaabe from The Anishinaabe of Wauzhushk Onigum, located in the northern shores of Lake of the Woods. Currently, Wabshkigaabo has begun to speak more openly about his lived experience living as a neurodivergent and Autistic First Nations man. He works to provide guidance to organizations and institutions on strength-based and culturally humble approaches when providing services for neurodivergent First Nations people. Dr. Carly McMorris is a cisgender white woman, an Associate Professor at the University of Calgary, and a Clinical Child Psychologist by training.

### Search strategy

The search terms were developed collaboratively with guidance from a health sciences librarian, with searches limited to English-language publications (see Table 1). Although eligibility was limited to Canadian contexts, broader Indigenous population terms from Canada, the United States, Australia, and international sources were retained at the search stage to maximize sensitivity in mapping a sparse and potentially inconsistently indexed field, which is consistent with scoping review methodology (28). During full-text review, records were eligible only when the population, setting, or analysis included Indigenous Peoples in Canada. Comprehensive searches were conducted in databases covering education, sport, Indigenous, and health sciences literature, including Medline, ERIC, Web of Science, CINAHL, SPORTDiscus, Bibliography of Indigenous Peoples in North America, iPortal, and Scopus. Grey literature was also explored through searches of ProQuest Dissertations and Theses, Canada Commons, Google Scholar, and through citation chaining to ensure thorough coverage.

**Table 1:** Full search strategy terms used across databases.

Concepts	Medline	EBSCO (ERIC, SPORTDiscus, CINAHL, Bibliography of Indigenous Peoples in North America)	Scopus, Indigenous Collection (Informit)	Web of Science, Google Scholar, Grey literature (Canada Commons, ProQuest Dissertations and Theses)
	Keywords	Keywords	Keywords	Keywords
	Title/Abstract	Title/Abstract	Title/Abstract	Title/Abstract
Concept 1 Indigenous people	1. Indian* 2. Native* 3. Inuit* 4. Aboriginal* 5. Metis 6. First Nation* 7. Indigenous Canadians^a^ 8. Native American 9. Pacific Islander 10. Torres Strait Islander	1. Indian* 2. Native* 3. Inuit* 4. Aboriginal* 5. Metis 6. First Nation* 7. Indigenous Canadians^a^ 8. Native American 9. Pacific Islander 10. Torres Strait Islander	1. Indian* 2. Native* 3. Inuit* 4. Aboriginal* 5. Metis 6. First Nation* 7. Indigenous Canadians^a^ 8. Native American 9. Pacific Islander 10. Torres Strait Islander	1. indigenous 2. Aboriginal 3. “first nations” 4. “American Indian” 5. “native American”
Concept 2 Neuro-developmental disorders	1. disabled person*/disabled people 2. mentally disabled person*/ mentally disabled people	1. disabled person*/disabled people 2. mentally disabled person*/ mentally disabled people	1. disabled person*/disabled people 2. mentally disabled person*/ mentally disabled people	1. disability 2. “intellectual disability” 3. “developmental disability” 4. “cognitive disability”
	3. developmental disab*	3. developmental disab*	3. developmental disab*	
	4. mental* retard*	4. mental* retard*	4. mental* retard*	
	5. activ* of daily living	5. cognitive impairment*	5. cognitive impairment*	
	6. functional limitation*	6. intellectual disabilit*	6. intellectual disabilit*	
	7. cognitive impairment*			
	9. intellectual disab*			
	10. neurodevelopmental disorder*			
	11. down syndrom*			
	12. autism			
	13. neurodevelopmt* 14. disabilit*			
	15. disable*			
Concept 3 Sport Recreation	1. motor activity	1. motor activ*	1. exercise	1. sport
	2. exercise	2. exercise	2. sport*	2. sports
	3. locomotion 4. sport* 5. exercise movement 6. physical education 7. running	3. locomotion 4. sport* 5. physical activ* 6. physical education 7. movement program*	3. “physical education” 4. “physical activit*” 5. “movement program*” 6. game*	3. athletes 4. “special Olympics” 5. “physical activity” 6. “physical education”
	8. walking	8. game*		
	9. physical fitness	9. physical fitness		
	10. play	10. play		
	11. names of sports^b^	11. names of sports^b^		

a Including specific names of First Nation identities (see Supplementary Material for full listing)

b Including specific names of sport activities (see Supplementary Material for full listing)

*Truncation symbol used to retrieve multiple word endings and variations. Search terms were adapted as appropriate for the indexing and controlled vocabulary of each database.

### Study selection

Following completion of the database searches, all retrieved references were imported into citation management software (Covidence), where duplicates were identified and removed. The remaining citations underwent a systematic two-stage screening process based on clearly defined inclusion and exclusion criteria. Initially, GB and SA independently screened titles and abstracts to determine preliminary eligibility. Articles identified as potentially relevant advanced to a full-text review, wherein the same reviewers independently assessed each article against the inclusion and exclusion criteria. Final inclusion decisions were made collaboratively through consensus between GB and SA.

### Inclusion and exclusion criteria

Literature was included if published from 2000 onward, addressing Indigenous individuals of any age with NDDs (including intellectual or developmental disabilities, cognitive disabilities, cerebral palsy, autism, fetal alcohol spectrum disorder, or related conditions) participating in sports, physical activity, or recreational programs within school or community contexts. Literature published prior to 2000 was excluded to ensure contemporary understandings, recognizing the significant evolution in discourse, language, and ethical standards relating to both NDDs and Indigenous perspectives. Articles were also excluded if they were news reports, opinion pieces, or if they lacked sufficient detail regarding Indigenous identity, neurodevelopmental disability, or specific involvement in sports or recreation to determine eligibility. While the primary focus was on Indigenous terms specific to Canada (First Nations, Métis, Inuit), international Indigenous terms (e.g., Pacific Islander, Native American) were also included to capture globally collaborative studies relevant to the Canadian context.

## Results

**Figure 1:**
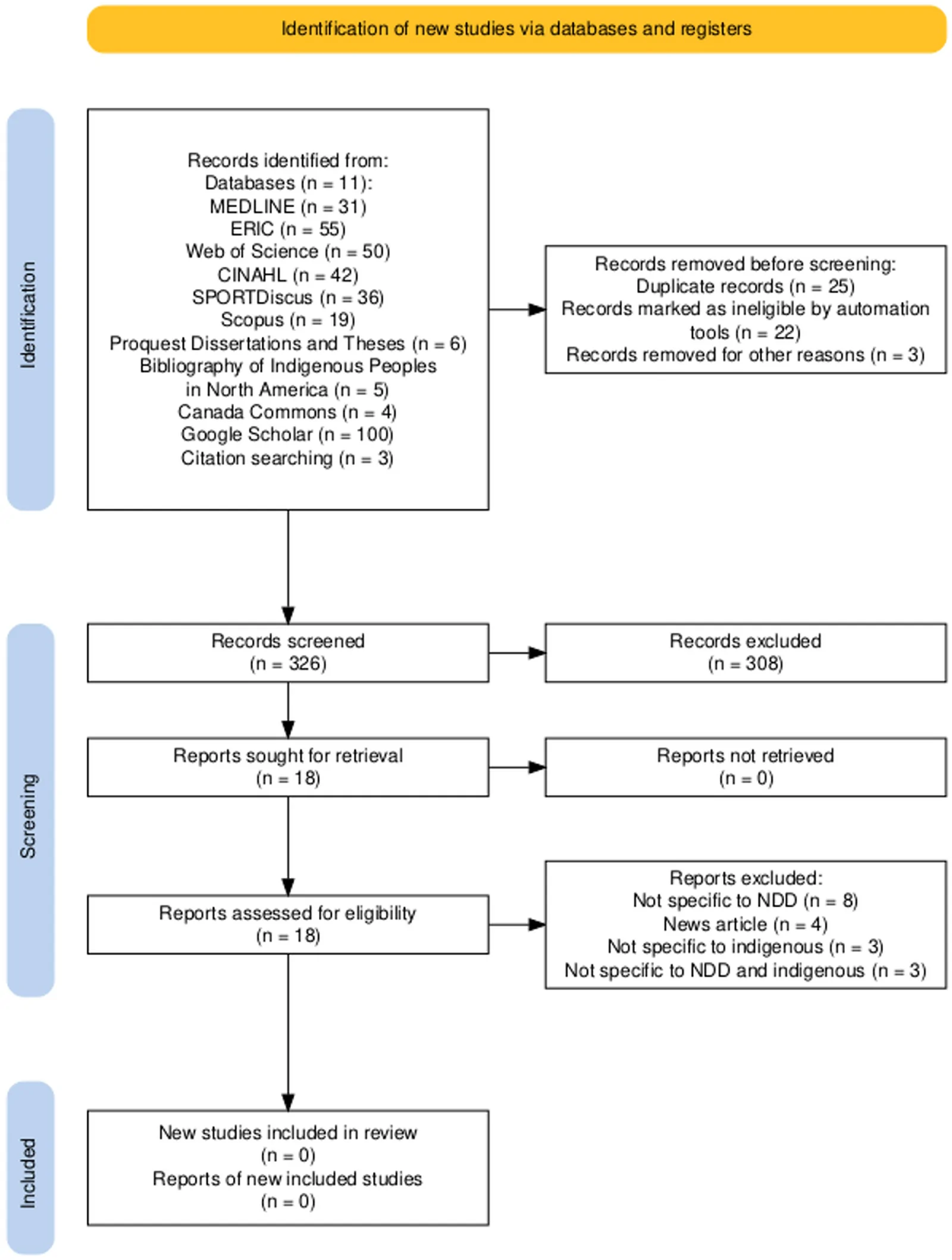
PRISMA 2020 flow diagram showing the study identification, screening, and eligibility process. Adapted from the PRISMA 2020 flow diagram template and populated with data specific to this review. The source is Page et al. (54). The template is licensed under the Creative Commons Attribution 4.0 International License (CC BY 4.0

The search strategy identified 326 records (see Figure 1). After duplicates were removed and titles and abstracts were screened, 18 records were retained for full-text review. All 18 full-text records were subsequently excluded. Four were not related to disability (32–35), four focussed only on physical disabilities, without addressing cognitive disability or NDDs (36–39), four were news stories that provided no empirical data or analysis (40–43), and three articles did not consider Indigenous people in their studies (44–46). Finally, three articles discussed individuals with NDDs and Indigenous people separately, but did not specifically mention NDDs in Indigenous people together (47–49). Consequently, no studies were eligible for inclusion in the current review.

## Discussion

The objective of this scoping review was to examine published and grey literature in physical activity in sport and recreation for Indigenous people with NDDs in Canada. The review identified no studies eligible for inclusion. Reviews, such as ours, that identify no eligible studies are often referred to as empty reviews, and though they do not permit synthesis of the included studies findings, they can still identify important adsences in the literature and clarify priorities for future research (50, 51). A total of 18 articles were screened for full-text review (47–49) that included both Indigenous Peoples, sport and recreation, and NDDs; however, they did not meet the inclusion criteria in any significant way or were not located within Canada.

### Research gaps in the evidence base

Although, Sutherland (2022) indicates there is a substantial gap in “academic studies on any area related to Indigenous Peoples in Canada with a disability” (49). Consistent with this, the absence of eligible studies in this empty review suggests that Indigenous Peoples with NDDs remain largely invisible in research related to sport and recreation, which may contribute to their continued exclusion in policy, programming, practice, and research. This absence may reflect structural conditions that shape which forms of knowledge are funded, published, and recognized as evidence. Colonial research structures have historically privileged Western biomedical and deficit-based understandings of disability, while Indigenous knowledge systems, community-led research, land-based activities, and relational, strengths-based approaches to health are underrepresented in conventional academic databases. This colonial exclusion of Indigenous knowledge is consistent with broader Indigenous disability scholarship, namely in Australia and Canada, which has identified the marginalization of Indigenous understandings of disability within dominant research frameworks (1, 5, 6, 12, 27). The absence of eligible literature, therefore, suggests a structural gap in knowledge production and in the recognition of Indigenous disability knowledge in sport and recreation research. This finding aligns with related literature showing that Indigenous disability, Indigenous sport and recreation, and disability-inclusive sport are often examined as distinct areas of inquiry. Scholarship has addressed Indigenous disability in broader contexts (1, 5, 6, 12, 27), Indigenous sport and cultural resurgence (10–12), and disability-inclusive sport (2, 8, 21, 22, 36); however, these areas rarely converge around Indigenous Peoples with NDDs in Canada. The absence of eligible studies therefore reflects a specific intersectional gap.

### Policy considerations

While it may appear that an empty review offers little evidence for synthesis, empty reviews can provide valuable insights by identifying absences in the evidence base, clarifying the scope of existing literature, and establishing priorities for future inquiry (50, 51). In the present review, the absence of eligible Canadian studies examining sport and recreation for Indigenous Peoples with NDDs should not be interpreted as indicating that this area is unimportant; rather, it suggests a structural policy gap in understanding how disability inclusion, Indigenous self-determination, and culturally grounded sport and recreation are connected in policy and implementation. The Truth and Reconciliation Commission's Calls to Action related to sport and reconciliation and Article 30 of the United Nations Convention on the Rights of Persons with Disabilities provide relevant policy anchors, but the lack of eligible literature indicates a need for research examining how these commitments are interpreted, implemented, and evaluated in community, school, and sport organization contexts (20, 26). Considered alongside related scholarship and policy frameworks, this absence suggests that policy research has not yet adequately addressed the intersection of Indigenous identity, NDDs, and equitable access to sport and recreation in Canada (8, 20, 25, 26).

### Practice and programming

In practice, addressing this gap requires the development of neuro-inclusive, community-driven programs that are co-created with Indigenous Peoples and rooted in Indigenous worldviews of disability and health. For example, a land-based physical activity program designed in partnership with an Indigenous community could integrate traditional games, storytelling, ceremony and other cultural teachings to support the physical, mental, emotional, and spiritual wellbeing of children and youth with NDDs. This would involve planning the initiative with Elders/knowledge keepers, families, and service providers, ensuring the activities are accessible, culturally safe, and responsive to the unique strengths and needs of participants. Rather than focussing on the deficits, the activities would centre on relationships, belonging, and connection to land and identity. The research on these types of programs should be led by, or meaningfully co-developed, by Indigenous scholars and community members using collaborative methods that uphold the principles of reciprocity, respect, and relational accountability (52). These approaches not only create meaningful opportunities for inclusion, but also help shift narratives toward self-determination in the health and well-being of Indigenous Peoples with NDDs. Collaborative research methods in the NDDs space have also been a priority for many and aligns with the call to action, “nothing about us, without us”. Future research in this area should be collaborative and community-driven, with Indigenous scholars, families, and communities taking lead in shaping the research. This will ultimately ensure that the resulting programs are culturally relevant, accessible, and grounded in Indigenous worldviews.

### Implications

The current state of published and grey literature on sport and recreation for Indigenous Peoples with NDDs in Canada is characterized by an absence of eligible studies. This empty review suggests three interrelated structural gaps: a research gap in the production and recognition of intersectional Indigenous disability sport and recreation knowledge; a policy gap in understanding how reconciliation, disability rights, and sport inclusion frameworks are applied to Indigenous Peoples with NDDs; and a practice gap in the evaluation of culturally grounded, neuro-inclusive sport and recreation programming. These gaps are suggested by the absence of eligible literature and by adjacent scholarship, rather than by findings from included studies (53).

## Conclusion

While it is widely recognized NDDs exist within Indigenous populations (27), this scoping review found no eligible Canadian literature examining the meaningful participation, access, or experiences of Indigenous individuals with NDDs in sports and recreation in Canada. This gap highlights a significant oversight in the literature and reflects a broader systemic neglect of Indigenous perspectives on disability, sports, recreation and inclusion. The absence of research in this area underscores the urgent need to build evidence that centres Indigenous voices and worldviews, recognizes the strengths and challenges of families and communities, and supports culturally rooted, accessible, and inclusive sports and recreation opportunities for Indigenous people with NDDs. Policymakers and funding agencies should take immediate action to fund and prioritize research that focuses on the intersection of NDDs, sport, and Indigenous populations. This will ensure the development of inclusive, culturally safe programs and policies that meet the needs of Indigenous Peoples with NDDs. Overall, this review provides a compelling rationale for future research, policy, and program development that addresses this overlooked yet significant need.

## Data Availability

The original contributions presented in the study are included in the article/Supplementary Material, further inquiries can be directed to the corresponding author.
